# Live Birth of a Healthy Child in a Couple with Identical mtDNA Carrying a Pathogenic c.471_477delTTTAAAAinsG Variant in the *MOCS2* Gene

**DOI:** 10.3390/genes14030720

**Published:** 2023-03-15

**Authors:** Maria Tofilo, Natalia Voronova, Leila Nigmatullina, Elena Kuznetsova, Valeria Timonina, Bogdan Efimenko, Oybek Turgunkhujaev, Svetlana Avdeichik, Muhammad Ansar, Konstantin Popadin, Anastasia Kirillova, Ilya Mazunin

**Affiliations:** 1 Center for Molecular and Cellular Biology, Skolkovo Institute of Science and Technology, 121205 Moscow, Russia; 2 Medical Genomics, 170100 Tver, Russia; 3 Fomin Clinics, 119192 Moscow, Russia; 4 School of Life Sciences, Ecole Polytechnique Fédérale de Lausanne, 1015 Lausanne, Switzerland; 5 Center for Mitochondrial Functional Genomics, Immanuel Kant Baltic Federal University, 236041 Kaliningrad, Russia; 6 Neurology Department, Semeynaya Clinic, 121059 Moscow, Russia; 7 A.I. Burnazyan Federal Medical and Biophysical Center, 123098 Moscow, Russia; 8 Department of Ophthalmology, Jules Gonin Eye Hospital, Fondation Asile Des Aveugles, University of Lausanne, 1015 Lausanne, Switzerland; 9 Advanced Molecular Genetics and Genomics Disease Research and Treatment Centre, Dow University of Health Sciences, Karachi 74200, Pakistan; 10 Swiss Institute of Bioinformatics, 1015 Lausanne, Switzerland

**Keywords:** *MOCS2* gene, Ohtahara syndrome, PGT-M, mtDNA relatives, consanguinity, maternal ancestry

## Abstract

Molybdenum cofactor deficiency type B (MOCODB; #252160) is an autosomal recessive metabolic disorder that has only been described in 37 affected patients. In this report, we describe the presence of an in-frame homozygous variant (c.471_477delTTTAAAAinsG) in the *MOCS2* gene in an affected child, diagnosed with Ohtahara syndrome according to the clinical manifestations. The analysis of the three-dimensional structure of the protein and the amino acid substitutions suggested the pathogenicity of this mutation. To prevent transmitting this mutation to the next generation, we used preimplantation genetic testing for the monogenic disorders (PGT-M) protocol to select *MOCS2* gene mutant-free embryos for transfer in an in vitro fertilization (IVF) program. As a result, a healthy child was born. Interestingly, both parents of the proband shared an identical mitochondrial (mt) DNA control region, assuming their close relationship and thus suggesting that both copies of the nuclear rare variant c.471_477delTTTAAAAinsG may have been transmitted from the same female ancestor. Our estimation of the a priori probability of meeting individuals with the same mtDNA haplotype confirms the assumption of a possible distant maternal relationship among the proband’s direct relatives.

## 1. Introduction

Molybdenum cofactor deficiency type B (MOCODB, #252160) is an inherited metabolic disorder that is characterized by a range of brain abnormalities, including hemimegalencephaly, agenesis of the corpus callosum, porencephaly, agenesis of the mammillary bodies, and dentato olivary dysplasia. In addition to these abnormalities, patients with MOCODB may also experience hypoxic injury, cortical dysplasias, and cerebral migration disorders [1]. The prognosis for individuals with MOCODB is generally poor, regardless of treatment, with only limited evidence to support the use of specific antiepileptic drugs.

The *MOCS2* gene encodes molybdenum cofactor and is located on chromosome 5q11.2. A mutation in the *MOCS2* gene can cause an early infantile epileptic encephalopathy, also known as Ohtahara syndrome. This condition was first described in 1976 by Ohtahara et al. (1976) in very young infants with specific electroencephalographic changes, and it is characterized by both clinical and electroencephalographic findings. Ohtahara syndrome is typically distinguished from early myoclonic encephalopathy by its presentation and imaging findings [2].

In this report, we describe a case of Ohtahara syndrome caused by a homozygous in-frame mutation in the *MOCS2* gene. After identification of the mutation, we were able to perform the PGT-M program, which resulted in the successful birth of a mutation-free baby. Modern medicine is unable to treat or somehow change the clinical course of Ohtahara syndrome. Thus, it is of most importance to timely select couples at risk of having a baby with this syndrome and prophylactic procedures. Thanks to genetic analysis technologies that are available in the world today, including whole exome sequencing and PGT-M, we have been able to identify an inherited disease that has been hidden from several families for years. We hope that the discovery of this new genetic variant will help other families to find the cause of their relatives’ condition.

## 2. Material and Methods

### 2.1. Medical History

**Figure 1:**
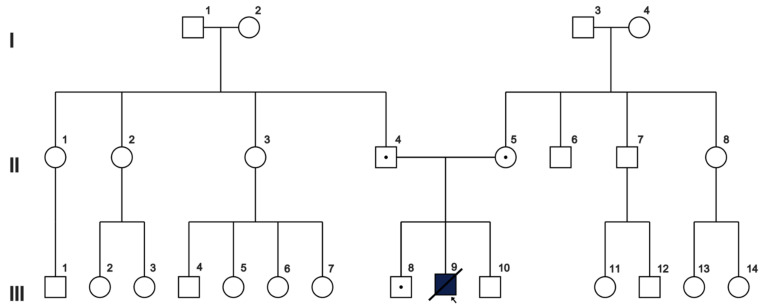
Pedigree of the family under study. The affected individual III9 is homozygous for the paternal c.471_477delTTTAAAAinsG and the maternal c.471_477delTTTAAAAinsG *MOCS2* mutations.

The married couple was referred to our clinic seeking fertility treatment due to having an affected child. The child was born as a result of a second pregnancy, with a high risk of miscarriage due to intrauterine infection at 23 weeks of gestation (placentitis by ultrasound examination). A vaginal delivery of a boy happened at 39 weeks of gestation with a weight of 3950 g. Postnatal adaptation was normal, and the APGAR score was 8/9. While the manuscript was being prepared, the child died. The couple claimed not to be related by blood (Figure 1). From the first day of his life, the child experienced daily myoclonic seizures, and there were no recorded perinatal insults. Diagnosis of Ohtahara syndrome was established based on the onset of myoclonic seizures before 3 months of age, clinical phenomenology of seizures, severe cognitive and motor delay, EEG findings of burst suppression pattern at 2 months, and MRI findings (corpus callosum agenesis, leukomalacia, cortical atrophy). From the age of 6 months, he had several epileptic seizures during respiratory viral infections. At the age of 1.5 years, he developed tonic asymmetric spasms with painful trunk flexion that lasted between 10 and 30 min. The frequency of tonic seizures continued to increase, with up to 30 attacks per day occurring at any time of the day, despite symptomatic treatment.

According to medical records, the child’s first EEG, performed at 2 months old, showed a suppression-burst pattern that persisted until he was 11 months old. However, at the age of 1.5 years, the EEG pattern changed to regional epileptic activity in the left fronto-anterior temporal leads, with spread to neighbouring areas and secondary synchronization but no burst-suppression pattern. A brain MRI performed when the child was 1 month old revealed complete agenesis of the corpus callosum, severe hemispheric cortical atrophy, severe diffuse hemispheric leukomalacia, hemispheric porencephaly, normal brainstem, and right hemispheric cerebellar hypotrophy. A neurological examination performed when the child was 4 years old revealed microcephaly and severe psychomotor retardation.

The child was unable to make productive verbal contact but responded to emotions. He was not able to track visual objects and had convergent strabismus. He also had poor swallowing and saliva drooling, as well as spastic tetraparesis, which meant he had no control over his body. He could not hold his head, flip over, or sit independently, and he had no control over his bowel or bladder movements. According to medication records, the child’s seizures were resistant to treatment with valproic acid, carbamazepine, clonazepam, and oxcarbazepine, either alone or in combination. Only levetiracetam and intravenous diazepam provided some decrease in the frequency of attacks.

### 2.2. Searching the Mutation

Whole exome sequencing (WES) was performed on the proband’s genomic DNA using the HiSeq4000 System (Illumina, San Diego, CA, USA). The results showed that the individual is homozygous for a variant, c.471_477delTTTAAAAinsG, on the paternal and maternal alleles of the *MOCS2* gene. We performed Sanger sequencing analyses to verify the mutation found by NGS method. Additionally, we verified the heterozygous status of the mutation by RFLP analysis using the DraI restriction endonuclease.

### 2.3. Crystal Structures Analysis and Pathogenicity Prediction

The three-dimensional structure of the wild-type of Mocs2 protein was downloaded from PDB (PDB accession code: 4ap8), and mutant Mocs2 3D structure was constructed with AlphaFold v2.3.1 [3]. The residual changes between the wild-type and mutant Mocs2 proteins were mapped to the model using PyMol v2.4 software. The pathogenicity of the mutations was predicted using multiple prediction software, including PROVEAN (http://provean.jcvi.org/ (accessed on 10 March 2023)), MutationTaster (http://www.mutationtaster.org/ (accessed on 10 March 2023)), CADD (https://cadd.gs.washington.edu/ (accessed on 10 March 2023)), and MutPred-Indel (http://mutpredindel.cs.indiana.edu/ (accessed on 10 March 2023)).

### 2.4. IVF Program

Controlled ovarian stimulation was performed for 11 days using Gonal-F (Merck Serono, Switzerland) and Menopur (Ferring, Germany). Human chorionic gonadotropin (HCG) 10,000 IU was used to trigger final oocyte maturation. During the oocyte pick-up, 18 cumulus-oocyte complexes were obtained. Conventional in vitro fertilization (IVF) was performed using the husband’s sperm, which had normal parameters. Thirteen oocytes were successfully fertilized and formed 10 blastocysts on days 5 and 6. Trophectoderm cells were biopsied from each blastocyst (5–10 cells per blastocyst) and sent for preimplantation genetic testing.

### 2.5. PGT-M and PGT-A

Whole genome amplification (WGA) was performed using VeriSeq PGS Kits (Illumina, San Diego, CA, USA) according to the manufacturer’s protocol. The WGA product was used for both PGT-A and PGT-M protocols. PGT-M was performed using Sanger sequencing and RFLP analysis on the WGA product. Next-generation sequencing reads were conducted on the MiSeq System (Illumina, San Diego, CA, USA) and visualized using the Bluefuse Multi Software (Illumina, San Diego, CA, USA).

### 2.6. mtDNA D-Loop Sequencing and Analysis

The mitochondrial DNA D-loop was sequenced using the Sanger method. The obtained chromatograms were analyzed using SnapGene software version 3.3.2.

The 55,180 annotated human mitochondrial genomes were downloaded from GenBank, and D-loop sequences were extracted from records. Pairwise comparison of all possible D-loop sequence pairs was conducted using module python-Levenshtein 0.20.9 with default distance weights parameters (1, 1, 1). Mitochondrial haplogroup was obtained using Haplogrep3 3.0.1 based on D-loop sequence. Sequences from GenBank were also annotated using Haplogrep3.

## 3. Results and Discussion

The first mention of the c.658_664delTTTAAAAinsG mutation was in 2003 when Reiss and Johnson referred to three Turkish patients who had the mutation as unpublished data [4]. The next and last mention of this particular mutation was in 2022 when Spiegel et al. published a comprehensive review of molybdenum cofactor deficiency. In this paper, the c.658_664delTTTAAAAinsG mutation was classified using a novel classification system and was referred to as the c.471_477delTTTAAAAinsG [1].

**Figure 2:**
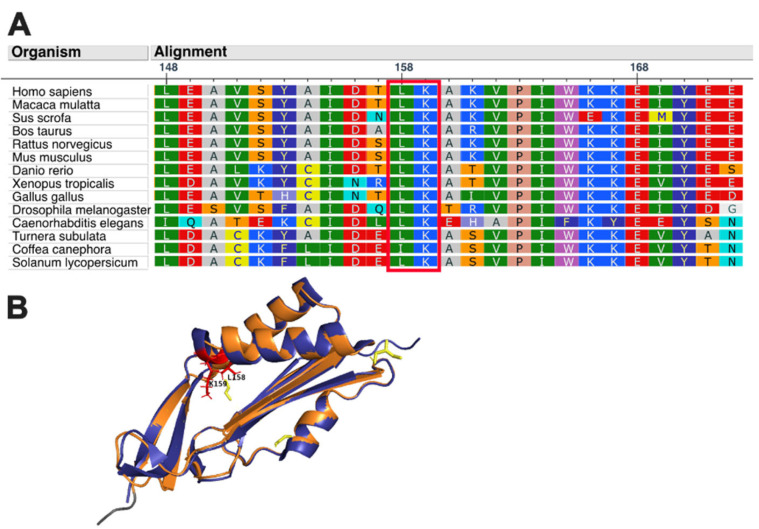
(**A**) Leucine (L) and Lysin (K) at positions 158–159 of Mocs2b protein are highly conserved in various species (the box marks the amino acids of interest). (**B**) Homology models for Molybdopterin synthase catalytic subunit (Mocs2b) protein were generated using the crystal structures of Mocs2b protein (PDB accession code: 4AP8) as the template. The purple cartoon represents the wild-type protein, and the orange cartoon represents the mutant protein p.Leu158_Lys159del. Yellow molecules are ligands, and yellow dots represent polar contacts.

The c.471_477delTTTAAAAinsG variant of the Mocs2 protein has not been reported in the ExAC (http://exac.broadinstitute.org/ (accessed on 10 March 2023)) or gnomAD (http://gnomad.broadinstitute.org/about (accessed on 10 March 2023)) databases. However, the previously described c.472_477del variant [5] is present in both databases and has a frequency of 1.19 × 10^−5^ in gnomAD v.2.1.1 and 1.65 × 10^−5^ in ExAC v.1.0. Both of these mutations do not cause a frameshift and result in the same deletion of two amino acids (Leu158 and Lys159) in the protein product (p.Leu158_Lys159del). Amino acid sequence alignment reveals that Leu158 and Lys159 are highly conserved across different species (https://www.ebi.ac.uk/Tools/msa/muscle/ (accessed on 10 March 2023)), suggesting that mutations in this area are likely pathogenic (Figure 2A). This mutation causes the deletion of two amino acids at the end of the last helix of the catalytic subunit, which is predicted to shorten the helix (Figure 2B). It is located near the active site, which could potentially disrupt the catalytic activity of molybdopterin synthase.

After identifying the pathogenic mutation in the proband, we performed an in vitro fertilization (IVF) program with preimplantation genetic testing for monogenic disorders (PGT-M) in order to determine a mutation-free embryo for transfer. As a result of the IVF program, we obtained ten blastocysts suitable for trophectoderm biopsy. Genetic testing revealed that seven embryos harbored the c.471_477delTTTAAAAinsG mutation in the homozygous condition, two embryos were in the heterozygous condition, and one was without the mutation. For the healthy embryo, we also performed preimplantation genetic testing for aneuploidies (PGT-A), which confirmed that the tested embryo was euploid and suitable for embryo transfer. Embryo transfer of this embryo resulted in a live birth of a healthy boy. To confirm the absence of the c.471_477delTTTAAAAinsG mutation in the *MOCS2* gene in the boy’s DNA, we performed Sanger sequencing and restriction fragment length polymorphism (RFLP) analysis using his blood sample as a DNA source. Both analyses confirmed the absence of the mutation.

**Figure 3:**
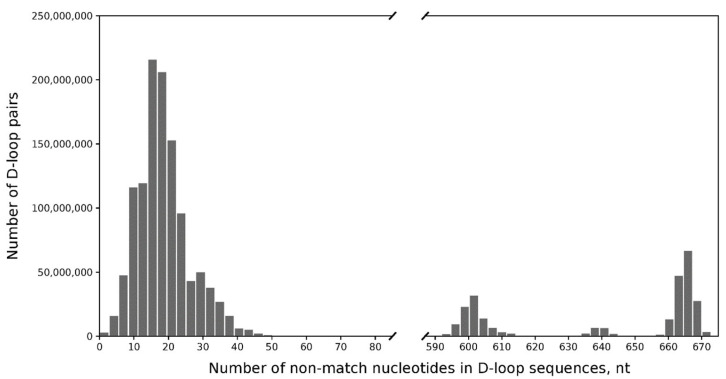
Distribution of mismatches and indels number in all possible pairs of D-loop sequences from the GenBank collection of 55,180 mitochondrial genomes.

Despite the couple’s claim that they are not closely related (at least not within two generations, according to their pedigree, as shown in Figure 1), it is a fascinating coincidence that they both carry the same nuclear DNA mutation. In this case, a comparison of short tandem repeat (STR) markers to determine kinship may not be successful due to the distant relationship between the husband and wife, as indicated by their pedigree. However, if the couple are distant maternal relatives, we would expect to see identical mitochondrial (mt) DNA sequences. To test this, we performed sequencing of the mtDNA D-loop and discovered that both parent’s and child’s samples are similar in their nucleotide changes (m.73A > G, m.195T > C, m.263A > G, m.315insC, m.499G > A, m.524insAC, m.16179C > T, m.16356T > C, m.16519T > C) and belong to U4 haplogroup according to Haplogrep3 annotation. Assuming a limited number of relatives in GenBank samples, we have not expected to find many pairs of similar mtDNA. We were interested in how often similar mtDNA could be found in annotated mtDNA sequences. A comparison of mitochondrial genomes from the GenBank showed the expected distribution of the number of mismatches and indels in random pairs of D-loop sequences (Figure 3). The median number of differences is 19 nucleotides, so for an arbitrary pair, we have expected to see that many differences. According to the distribution, 0 to 3 differences in D-loops are observed rarely, and the probability of observing identical D-loop sequences is 0.00022. This is why D-loop sequences with no differences are likely to mark close relatives. Moreover, precise analysis of the D-loop sequence mutations showed that in the GenBank, there are no such D-loop sequences, and among the most similar ones, there are only 38 sequences carrying seven out of eight mutations observed in the proband (all except m.524insAC) along with their own specific mutations. The rareness of a given nucleotide sequence in GenBank, as well as the a priori low probability of random meeting of such individuals, allowed us to assume that the couple may have a distant maternal relative (remote relative consanguineous marriage) and that the nuclear c.471_477delTTTAAAAinsG variant may have been transmitted from this female ancestor.

The well-known fact is that consanguineous marriage has negative consequences when it comes to the increased genetic risks for the offspring, as opposed to the potential social and economic benefits. This type of marriage is traditional and respected in many communities in North Africa, the Middle East, and West Asia. It is also practiced among emigrant communities from highly consanguineous countries and regions [6]. The custom of endogamous marriage among individuals belonging to the same clan or tribe often results in an unequal distribution of founder mutations across populations. Consanguinity may also affect fertility rates and pregnancy outcomes, such as increased pregnancy loss and preterm labor [7]. In addition, allelic heterogeneity for rare autosomal recessive disorders has been observed in an increasing number of highly consanguineous populations. The case presented here demonstrates that preimplantation genetic diagnosis and screening can and should be used to mitigate some of the adverse reproductive outcomes associated with consanguinity.

Traditionally, studies of consanguineous marriages are focused on the nuclear genome only, where rare homozygous variants located within the runs of homozygosity can cause disease. Here, we would like to emphasize also the utility of the mitochondrial genome: (i) if the pedigree is unknown or known incompletely, the mitochondrial comparison of parents can help to reconstruct the pedigree and trace back the origin (maternal or paternal ancestry) of the causative variant; (ii) large-scale future analyses of consanguineous families with causative variants inherited from maternal or paternal ancestors can uncover new properties of gender-specific mutagenesis [8]; (iii) in some cases, the mitochondrial genome, completely independent of consanguinity, can carry the deleterious causative variant [9].

## Data Availability

The data supporting this study’s findings are available from the corresponding author upon reasonable request.
